# A13 THE RATE OF ADVERSE EVENTS AFTER COVID-19 VACCINATION IS SIMILAR IN PATIENTS WITH CELIAC DISEASE AND NON-CELIAC POPULATION: RESULTS OF A LARGE INTERNATIONAL CROSS-SECTIONAL STUDY

**DOI:** 10.1093/jcag/gwac036.013

**Published:** 2023-03-07

**Authors:** S Tandon, J P Stefanolo, L Russell, M D L Paz Temprano, S Niveloni, E Verdu, D Armstrong, B Lebwohl, D Leffler, J Tye-Din, A Day, C Olano, V Lopez, L Uzcanga, E Madaria, M Montoro Huguet, S Vivas, A Rodriguez-Herrera, G Makharia, D Sanders, J Zeitz, C Mulder, C Ciacci, F Valerio, M I Pinto-Sanchez

**Affiliations:** 1 Health Sciences, McMaster University, Hamilton, Canada; 2 Hospital B Udaondo, Buenos Aires, Argentina; 3 Medicine, McMaster University, Hamilton, Canada; 4 Columbia University, New York; 5 Gastroenterology, Harvard University, Boston, United States; 6 Immunology, Melbourne University, Melbourne, Australia; 7 Paediatric Research, University of Otago, Christchurch, New Zealand; 8 Universidad de la Republica, Montevideo, Uruguay; 9 Instituto Nacional de Ciencias Médicas y Nutrición Salvador Zubirán, Mexico City, Mexico; 10 Hospital General Universitario de Alicante, Alicante; 11 Unidad de Gastroenterología, Hepatología y Nutrición, Huesca; 12 Universidad de Leon, Leon, Spain; 13 St Luke's General Hospital, Kilkenny, Ireland; 14 All India Institute of Medical Sciences, New Delhi, India; 15 Infection, Immunity and Cardiovascular Disease, The University of Sheffield, Sheffield, United Kingdom; 16 Hirslanden Group, Zurich, Switzerland; 17 Amsterdam UMC, Amsterdam, Netherlands; 18 Medicine and Surgery, Università degli Studi di Salerno, Baronissi, Italy; 19 Albert Einstein Hospital Israelita, Sao Luiz, Hospital Sirio Libanes, Sao Paulo, Brazil

## Abstract

**Background:**

Patients with celiac disease (CeD) reported increased COVID-19 vaccine hesitancy due to a fear of adverse events (AEs). However, the risk of AEs post-COVID-19 vaccination in patients with CeD is unknown.

**Purpose:**

To assess whether the rate of common side effects (SEs) and AEs due to COVID vaccines are higher in patients with CeD compared to a non-CeD population.

**Method:**

We conducted a collaborative international cross-sectional study in 16 countries between April 2022 and July 2022. An online survey was distributed to patients with CeD through patients’ local societies, and to non-CeD from the general population in each country through social media posts, word-of-mouth, and through academic institutions. We collected data on participant demographics, medical conditions, CeD diagnosis, GFD adherence, history of COVID-19 vaccinations (type and doses) and self-reported SEs and AEs post-COVID-19 vaccine. SEs included pain/swelling at the site, fatigue, fever, chills, nausea and/or headaches. AEs included thrombosis, myocarditis, anaphylactic reaction, and hospitalization related to the vaccine. Logistic regression models were used to assess predictors such as CeD diagnosis, age, gender, vaccine type and comorbidities on the likelihood of reporting SEs and AEs post-vaccine.

**Result(s):**

: A total of 17,795 participants completed the survey, 13,638 with CeD (median age of 45[27]) and 4,157 non-CeD controls (median age of 43[20]). There were no significant differences in sex between CeD and controls. Overall, CeD patients had similar odds of SEs compared with non-CeD individuals (aOR=1.02;95% CI=0.92-1.14). SEs were slightly increased only in the second dose of the vaccine in the CeD population compared to non-CeD individuals (aOR= 1.35; 95% CI=1.19-1.53). The most common reported SEs in CeD and controls were pain/swelling at the injection site (29% vs 23 %, p< 0.0001) and fatigue (29% vs 24%, p<0.0001). The odds of SEs were higher with Moderna Spikevax, AstraZeneca/Oxford and Johnson and Johnson vaccines than after the Pfizer vaccine (p< 0.0001). The overall rate of AEs post-vaccine was similar between patients with CeD and non-CeD individuals (aOR= 1.29; 95% CI= 0.89-1.87). Overall, female gender, older age, GFD adherence, respiratory conditions, obesity and receiving immunosuppressive medications increased the odds of SEs, while only age and a history of allergies increased the odds of AEs.

**Conclusion(s):**

In this large international study, patients with CeD reported similar rates of SEs and AEs post-COVID vaccine compared to non-CeD individuals. This information is highly relevant as it addresses the main concern leading to COVID-19 vaccine hesitancy in CeD patients.

**Disclosure of Interest:**

None Declared

